# Development of a Cellular Membrane Nanovesicle-Based Vaccine Against Porcine Epidemic Diarrhea Virus

**DOI:** 10.3390/cells15020208

**Published:** 2026-01-22

**Authors:** Xianjun Wang, Weibing Zhang, Hong Hu, Wenjing Gao, Xu Ma, Yarong Wu, Yongfeng Qiao, Yang Wang, Ding Zhang, Chunbo Dong, Haidong Wang, Zhida Liu

**Affiliations:** 1 College of Veterinary Medicine, Shanxi Agricultural University, Jinzhong 030801, Chinadongchunbo@cpu.edu.cn (C.D.); 2 Shanxi Academy of Advanced Research and Innovation, Taiyuan 030032, China; 3 School of Elite Biomedical Engineers, China Pharmaceutical University, Nanjing 210009, China; 4 School of Biopharmacy, China Pharmaceutical University, Nanjing 210009, China

**Keywords:** cellular membrane nanovesicle, vaccine, *porcine epidemic diarrhea virus*, S protein, immune evaluation

## Abstract

**Highlights:**

**What are the main findings?**

- A highly efficient veterinary vaccine platform was developed using cellular membrane nanovesicles (CMN) and validated for PEDV by presenting the spike protein.
- The CMN-based PEDV vaccine elicited robust humoral and CD8^+^ T cell immune responses in both murine and porcine models, accompanied by notable virus-neutralizing antibody production.

**What are the implications of the main findings?**

- The CMN-based platform enables multivalent PEDV vaccine design by co-expressing spike proteins from diverse viral variants to elicit broad-spectrum protection.
- The CMN-based approach provides a versatile strategy for the modular development of polyvalent and multivalent veterinary vaccines via co-expression of antigens from distinct virus.

**Abstract:**

*Porcine epidemic diarrhea virus* (*PEDV*) has emerged as a major pathogen responsible for porcine diarrheal diseases, causing outbreaks of severe diarrhea and high mortality in neonatal piglets, thereby inflicting severe economic losses on the global swine industry. Current commercial *PED* vaccines, comprising conventional inactivated and live attenuated formulations, have exhibited progressively diminished efficacy in the face of emerging *PEDV* variants. The development of high-efficiency vaccine platforms is therefore critical for *PED* control. This study engineered a cellular membrane nanovesicle (CMN)-based vaccine, which differs from existing inactivated or subunit vaccines by presenting the PEDV spike (S) protein on the cell membranes to mimic the bilayer phospholipid structure of the viral envelope. The full-length S protein (FS, aa 19-1309) or a truncated S protein fragment (TS, aa 19-726) was expressed in Expi293F cells, followed by extraction of cell membranes to assemble antigen-displaying CMN vaccines. Compared with commercial live attenuated vaccine, administration of the CMN vaccine elicited high-titer neutralizing antibodies and elevated IFN-γ-producing CD8^+^ T cells in murine studies. Safety assessments revealed no adverse effects on body weight, hepatic/renal function indices, or histopathological parameters in vaccinated mice. Furthermore, immunization of piglets elicited notable humoral and CD8^+^ T cell immune responses. Collectively, the strategy of CMN-based vaccine described herein delivers a potential *PEDV* vaccine platform, thereby offering a novel avenue for next-generation veterinary vaccine development.

## 1. Introduction

*Porcine epidemic diarrhea virus* (*PEDV*) is an important pathogen within the genus *Alphacoronavirus* within the family *Coronaviridae.* Since 2010, highly pathogenic variants of *PEDV* have caused large-scale outbreaks, characterized by high morbidity, high mortality, and widespread transmission. *PEDV* is transmitted primarily via the fecal–oral route and specifically infects the small intestinal epithelial cells of newborn piglets, leading to acute enteritis, villous atrophy, and malabsorption. Consequently, mortality in piglets can reach up to 100% [1,2,3,4]. The continued prevalence of *PEDV* poses a persistent threat to the global swine industry, resulting in substantial economic losses [5,6].

Phylogenetic analyses classify *PEDV* into two groups: GI (classical strains) and GII (variant strains). The GI group can be further subdivided into GIa and GIb sublineages. The GIa sublineage includes early European isolates such as *CV777* and *DR13*, while GIb consists mainly of cell-adapted strains. The GII group is divided into GIIa, GIIb, and GIIc sublineages. Vaccines developed based on classical GI strains like *CV777* provide limited immune protection against currently prevailing GII strains [7]. Current prevention and control strategies rely mainly on live attenuated and inactivated vaccines. However, live vaccines carry the risk of virulence reversion, while inactivated vaccines often elicit insufficient neutralizing antibody responses due to poor antigen presentation efficiency. Moreover, both exhibit limited cross-protection efficacy against prevailing strains [8,9]. Therefore, in light of the current epidemiological status and the limitations of existing control strategies, there is an urgent need to develop novel vaccine candidates to reduce the infection risk in neonatal piglets and achieve effective prevention and control of *PEDV* variants.

*PEDV* is an enveloped virus with surface projections, measuring approximately 95–190 nm in diameter. Its genome consists of a single-stranded positive-sense RNA molecule approximately 28 kb in length, which includes a 5′ untranslated region (UTR), open reading frames (ORFs), and a 3′ UTR. The ORFs encode four structural proteins: the membrane (M) protein, nucleocapsid (N) protein, envelope (E) protein, and spike (S) protein. The M protein is a highly conserved transmembrane protein composed of 227 amino acids. Its M3 domain (residues 160–226) exhibits strong immunogenicity and has been identified as the major immunodominant region of the M protein [10,11]. The N protein, comprising 441 amino acids, is also highly conserved. During the early stages of infection, high levels of antibodies against the N protein are produced in pigs, making it a potential diagnostic marker for early PEDV detection [12]. The E protein is a small envelope glycoprotein with a molecular mass of approximately 8 kDa, consisting of 76 amino acids. It demonstrates relatively poor antigenicity [13]. In contrast, the S protein, a trimeric glycoprotein uniquely present on the viral surface, has been extensively studied in recent years. It not only mediates receptor binding and membrane fusion but also serves as a key target for the induction of potent neutralizing antibodies [14,15].

In recent years, in addition to inactivated and attenuated vaccines, various types of novel vaccine platforms have been continuously explored and developed. Truncated forms of the PEDV S protein were expressed using a eukaryotic expression system and the antibody titers induced by these *PEDV* subunit vaccines were further evaluated. The results showed that the PEDV S protein exhibits potent immunogenicity [16]. Another study reported a PEDV S protein-based mRNA vaccine, which triggered effective antibody responses and specific T cell reactions in immunized piglets [17]. However, the development of S protein-based vaccines still faces considerable challenges, such as complex glycosylation patterns and manufacturing processes. Although mRNA vaccines encapsulated in lipid nanoparticles (LNP) have achieved high rates of protection in challenge studies, their clinical use is hampered by inherent instability of mRNA and stringent cold-chain requirements [18,19,20]. Given the current prevalence of *PEDV* and the limitations of existing vaccine technologies, there is a pressing need to develop a versatile veterinary vaccine platform that features simple production processes and suitability for large-scale applications.

Notably, the technology of CMN has demonstrated unique advantages. It was confirmed that nanoparticles coated with tumor cell membranes can mimic the membrane structure of pathogens, enhance antigen presentation efficiency, and significantly strengthen T-cell immune memory [21]. This technology leverages the natural cell membrane’s phospholipid bilayer structure and membrane surface functional proteins to simulate the membrane fusion mechanism during viral infection, thereby promoting efficient endocytosis and cross-presentation by dendritic cells (DCs) while inducing robust humoral and cellular immunity [22,23,24]. These advances in cancer research suggest the potential of applying the CMN technology to the development of veterinary vaccine platforms. In this study, we introduce the CMN technology into the field of *PEDV* vaccine development. This approach offers a platform to address the efficacy and safety concerns associated with conventional vaccines while also establishing a theoretical foundation for next-generation veterinary vaccine development.

## 2. Materials and Methods

### 2.1. Cells, Virus and Animals

Vero cells (Thermo Fisher Scientific, Waltham, MA, USA, Cat #CCL-81) were cultured in Dulbecco’s Modified Eagle Medium (DMEM; Gibco, Grand Island, NY, USA, Cat #11995065) supplemented with 10% fetal bovine serum (FBS; Gibco, Cat #A5256501). Expi293F cells (Thermo Fisher Scientific, Cat #A14527) were maintained in SMM 293-TII medium (BGI, Shenzhen, China, Cat #M293TII). Both cell lines were incubated at 37 °C under 5% CO_2_, with Expi293F cells cultured in a shaking incubator. The PEDV GIIa strain was isolated and preserved in our laboratory, and the virus was propagated in Vero cells.

Six to eight-week-old female BALB/c mice were purchased from Beijing Vital River Laboratory Animal Technology Co., Ltd. Thirty-day-old specific-pathogen-free (SPF) ternary hybrid piglets were supplied by Tianyu Biology. All animals were housed in a SPF environment at the animal care facility of Shanxi Agricultural University. Experimental procedures were strictly performed in compliance with the guidelines of the Institutional Animal Care and Use Committee of Shanxi Agricultural University (SXAU-EAW-2023M.DF.001017216/2024P.FU.0050223856).

### 2.2. Plasmid Construction and Transfection

To establish a CMN vaccine against *PEDV*, we selected two immunogens based on the S protein sequence from the prevalent GIIa strain of PEDV: a full-length S protein construct (FS, aa 19-1309) and an N-terminal truncated S protein construct (TS, aa 19-726). Both genes were codon-optimized, synthesized (Logenbio, Shanghai, China), and subsequently cloned into the pCDH vector. Expi293F cells were transfected with constructed pCDH vector using polyethyleneimine (PEI; AbMole, Houston, TX, USA, Cat #M40823) at a plasmid to PEI ratio of 1:3 (1 μg plasmid/mL cells); the expression of S proteins was confirmed by flow cytometry and Western blot at 48 h post-transfection.

### 2.3. Production and Quantification of CMN Vaccines

The production of CMN was performed using a classical sub-cellular fractionation protocol. Forty-eight hours post-transfection, cells were harvested (2000 rpm, 5 min) and washed three times with TM buffer [0.01 M Tris (Solarbio, Beijing, China, Cat #T8060) and 0.001 M MgCl_2_ (Invitrogen, Carlsbad, CA, USA, Cat #AM9530G), pH 7.4]. Following three successive freeze–thaw cycles for hypotonic lysis, the pellet was resuspended in 0.25 M sucrose (Aladdin, Shanghai, China, Cat #S112228). Debris were removed (2000 rpm, 10 min, 4 °C), and the supernatant was then transferred to a new tube. Subsequently, it was centrifuged at 10,000 rpm for 30 min to pellet the desired components. The resulting membrane pellet was washed three times with PBS and then extruded sequentially through 800 nm, 400 nm, and 200 nm polycarbonate porous membranes using a mini extruder (Avanti Polar Lipids, Alabaster, AL, USA). The protein content was further measured by BCA assay (Beyotime Biotechnology, Shanghai, China, Cat #P0012). The size distribution was determined by nanoparticle tracking analysis (NTA) with a Nanoparticle Tracking Analyzer (Malvern Instruments, Worcestershire, UK). Then, the vaccine preparation was formulated by mixing the appropriate volume of CMN with Montanide™ GEL 02 PR adjuvant (Seppic, Paris, France, Cat #36084X) at a volume ratio of 9:1, followed by incubation at 4 °C for 1 h.

Quantification of PEDV S antigen in commercial or CMN vaccines was performed using ELISA with LHHG3-LK4G3 and LB9 antibodies, both of which are recombinant monoclonal antibodies consisting of the original mouse-derived variable regions fused to a human IgG1 Fc fragment. Briefly, ELISA plates were coated overnight at 4 °C with LHHG3-LK4G3 (2 μg/mL in PBS, pH 7.0). After blocking with 5% fat-free milk in PBS, sonicated vaccine samples and serially diluted recombinant PEDV S protein standards were added and incubated. Subsequently, a HRP-conjugated PEDV S-specific antibody (LB9-HRP) was applied. Color development was initiated using TMB substrate (Beyotime Biotechnology, Cat #P0209), stopped with 2 M H_2_SO_4_ (Solarbio, Cat #C1058), and absorbance was measured at 450 nm. The concentration of PEDV-S antigen in each vaccine sample was quantified based on the standard curve. This mass-based concentration was then converted to molar concentration by normalizing to the respective molecular weight of the antigens according to the formula [n (number of moles) = m (mass in grams)/M (molar mass)]. The resulting molar values were used as the basis for dosing in the subsequent vaccination studies.

### 2.4. Mouse Vaccination and Sample Collection

Female BALB/c mice were randomly assigned to different groups (n = 5 per group), and given intramuscular injections of the CMN-TS vaccine at ascending doses (0.95, 1.90, 3.80, 7.50, or 15.00 pmol per mouse), the CMN-FS vaccine (3.80 pmol per mouse), or a commercial vaccine (Wuhan Keqian Biology Co., Ltd., Wuhan, China; 3.80, 38.00, and 76.00 pmol per mouse). PBS was included as a vehicle control. Following the primary immunization, all vaccinated mice were boosted intramuscularly at weeks 2 and 4. Blood samples were collected one day before the second and third immunizations, and serum was isolated for endpoint ELISA to determine antibody titers. Seven days after the final booster, mice were euthanized, and spleens were harvested for lymphocyte isolation. T cell responses were subsequently assessed via IFNγ intracellular staining followed by flow cytometry analysis.

### 2.5. Piglets’ Vaccination and Sample Collection

Six piglets were randomly assigned to two groups (n = 3 per group) and given intramuscular injection of either vehicle (PBS control) or 100 pmol of the CMN-FS vaccine in the neck, respectively. Booster immunizations were administered at two and four weeks after the primary immunization. Serum samples were collected 13 days after the first two immunizations. Seven days after the final booster immunization, blood was collected to isolate lymphocytes for IFN-γ ELISPOT assay.

### 2.6. Enzyme-Linked Immunosorbent Assay (ELISA)

The ELISA plates were coated with recombinant PEDV S protein (2 μg/mL in PBS, pH 7.0). After blocking with 5% non-fat milk in PBS, plates were washed three times with PBST. Serially diluted mouse or piglet sera were then added and incubated. Bound IgG was detected with goat anti-mouse IgG-HRP (EasyBio, Pasig, Philippines, Cat #BE0102) or goat anti-pig IgG-HRP (Abcam, Cambridge, UK, Cat #ab6915) followed by development with TMB substrate. The reaction was terminated with 2 M H_2_SO_4_, and absorbance was read at 450 nm. The endpoint titer was calculated as the highest serum dilution that produced an OD value exceeding 2.5 times the background level.

### 2.7. Neutralization Assay

Vero cells were seeded into 96-well plates. Prior to the assay, serum samples were heat-inactivated at 56 °C for 30 min and subjected to two-fold serial dilution in cell culture medium. Each diluted serum was then mixed with 200 TCID_50_/50 μL of virus and incubated at 37 °C for 1 h. Subsequently, the serum–virus mixtures were transferred to the pre-seeded cell plates. Cytopathic effect (CPE) was monitored daily, and neutralizing antibody titers were calculated by the Reed–Muench method.

### 2.8. Flow Cytometry Analysis

At 48 h post-transfection, Expi293F cells were harvested and washed twice with PBS. The cells were then resuspended in PBS containing 2% FBS and incubated with an S-specific primary antibody (LHHG3-LK4G3), washed three times with PBS, and subsequently incubated with an APC-conjugated anti-human IgG secondary antibody. Following three additional washes, the surface expression of S proteins was determined by flow cytometry using a Beckman Coulter CytoFLEX instrument (Beckman Coulter, Inc., Brea, CA, USA).

Single-cell suspensions were generated from the spleens of immunized mice and then plated at 2 × 10^6^ cells per well. After stimulation with 10 μg/mL of PEDV S protein for 6 h at 37 °C in the presence of brefeldin A, cells were blocked with anti-FcγIII/II receptor (clone 2.4G2) and stained with fixable viability dye eFluor™ 450 (eBioscience, San Diego, CA, USA) and APC-conjugated anti-mouse CD8 α (BioLegend, San Diego, CA, USA) for surface staining. After being fixed and penetrated using the True-Nuclear^TM^ transcription factor buffer set (BioLegend), PE-conjugated anti-mouse IFN-γ (BioLegend) was applied for intracellular staining. Finally, the proportion of IFN-γ^+^ cells in CD8^+^ T cell populations were analyzed using a Beckman Coulter CytoFLEX flow cytometer (Beckman Coulter, Inc., Brea, CA, USA).

### 2.9. Enzyme-Linked Immunospot (ELISPOT) Assay

Peripheral blood mononuclear cells (PBMC) of piglets were isolated using a lymphocyte separation kit (TBD) according to the manufacturer’s instructions. The lymphocytes were then resuspended in RPMI-1640 (Gibco, Cat #11875093) medium supplemented with 10% FBS at a density of 1 × 10^5^ cells/mL and plated (200 µL/well) onto anti-IFN-γ–coated ELISPOT plates. Lymphocytes were stimulated with PEDV-S protein (10 μg/mL, produced and stored in-house previously) or PBS (negative control) and incubated for 24 h. Subsequently, spots corresponding to IFN-γ-secreting cells were then developed and enumerated according to the manufacturer’s protocol (Mabtech, Nacka Strand, Sweden).

### 2.10. In Vivo Toxicity Analysis

Following immunization, body weights were monitored daily for 14 days. Serum samples were collected 48 h after the booster immunization for the analysis of alanine aminotransferase (ALT), aspartate aminotransferase (AST), and creatinine (CREA). For histopathological evaluation, major organs (heart, liver, spleen, lungs, and kidneys) were harvested seven days after the booster immunization, followed by fixation and Hematoxylin and Eosin (H&E) histopathology.

### 2.11. Statistical Analysis

Data were presented as mean ± standard deviation (SD). Intergroup differences were assessed for statistical significance via mixed-effects analysis or one-way analysis of variance (ANOVA) combined with Tukey’s multiple comparison post-test. For comparisons between two groups, unpaired, two-tailed Student’s *t*-tests were applied. *p* < 0.05 was considered statistically significant, denoted as * *p* < 0.05, ** *p* < 0.01, *** *p* < 0.001, and ns—not significant.

## 3. Results

### 3.1. Preparation and Characterization of CMN Vaccines

**Figure 1:**
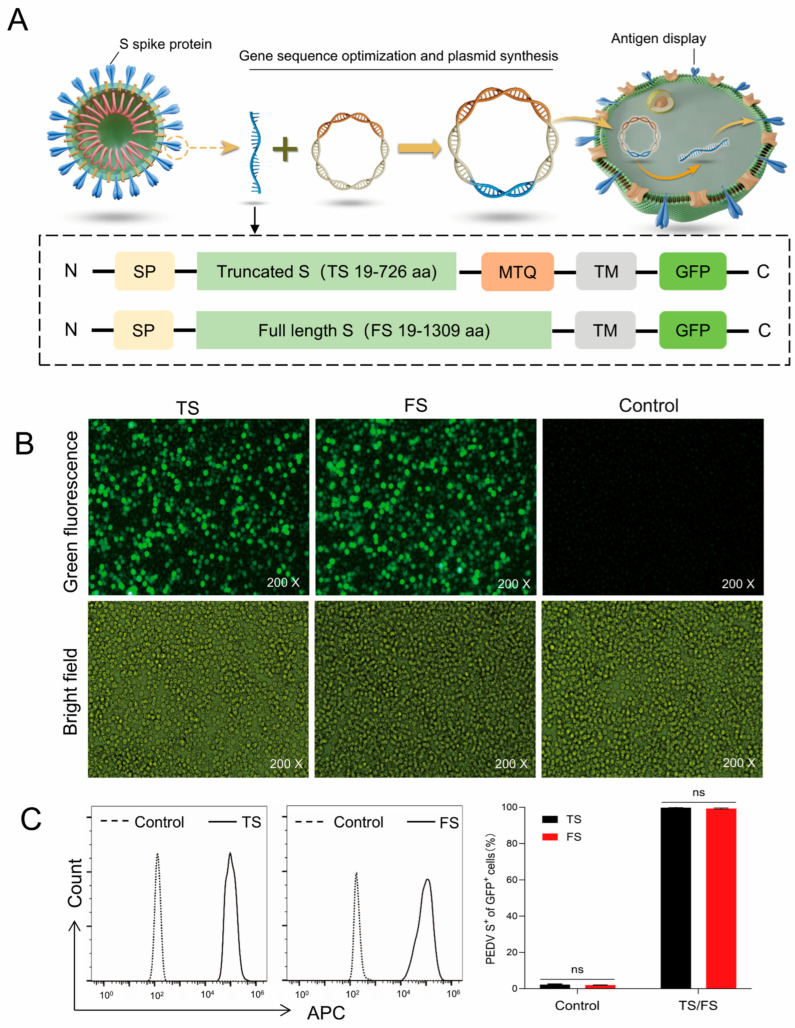
Design and expression validation of PEDV S protein. (**A**) Schematic diagram of the vector design and protein expression on membrane surface. FS: full-length S protein of PEDV; TS: truncated S protein of PEDV; SP: IgGκ signal peptide (sequence: METDTLLLWVLLLWVPGSTGD); MTQ: trimer tag; TM: transmembrane area. (**B**) Green fluorescence observation via fluorescence microscopy after 48 h of transfection. (**C**) Both GFP and APC signals were detected by flow cytometry after 48 h of transfection; S protein expression on GFP-positive cells was analyzed, using the S-specific antibody (LHHG3-LK4G3) as the primary antibody and an APC-conjugated anti-human IgG as the secondary antibody, with un-transfected Expi293F cells serving as control. (ns, not significant).

Given that the S protein, which consists of S1 and S2 subunits, represents the primary antigenic target for inducing neutralizing antibodies against *PEDV*, two recombinant candidate antigens were designed and constructed based on its functional structure: the full-length S protein (FS) and a truncated S protein (TS, S1). Both constructs were engineered to contain a C-terminal transmembrane anchor and an EGFP reporter for downstream detection and functional assays. Furthermore, a trimer tag was incorporated into the TS construct to enhance its trimerization efficiency (Figure 1A). To evaluate the expression of these immunogens in vitro, the corresponding recombinant plasmids were transiently transfected into Expi293F cells, and protein expression were assessed via GFP fluorescence (Figure 1B) and flow cytometry (Figure 1C). The results demonstrated successful expression of both FS and TS proteins, confirming the correctness of the vector design and the reliability of protein expression.

**Figure 2:**
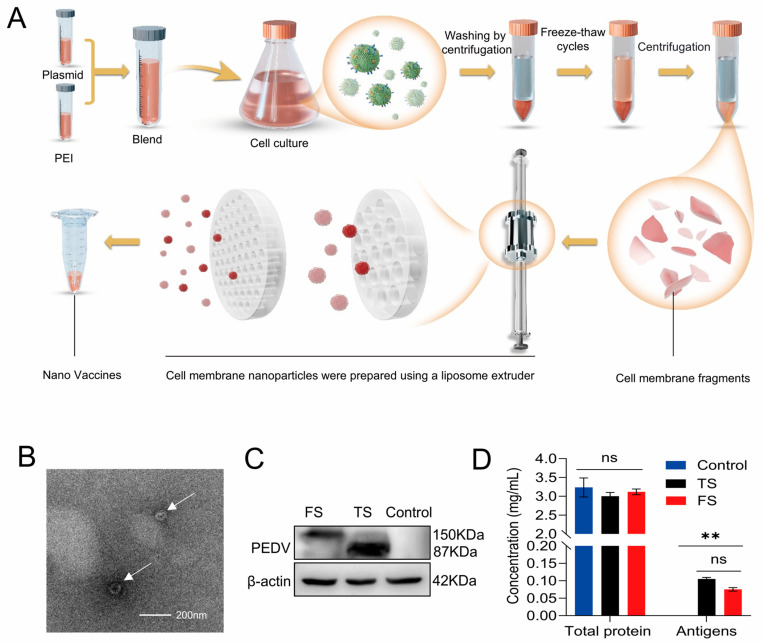
Preparation, characterization, and quantification of CMN vaccines. (**A**) Preparation process of CMN vaccine. (**B**) TEM image of a CMN vaccine. Scale bar, 200 nm. (**C**) Western blot analysis of FS and TS expression levels in the cell membrane fraction at 48 h after transfection. (**D**) ELISA quantification of FS and TS content in the CMN vaccines; CMN derived from un-transfected Expi293F cells served as control. (ns, not significant; ** *p* < 0.01).

To illustrate the preparation process of the CMN vaccines, a schematic workflow was provided (Figure 2A). Transmission electron microscopy (TEM) analysis revealed that the obtained nanovaccines exhibited typical nanoparticle-like structures (Figure 2B) and nanoparticle tracking analysis revealed a mean particle size of ∼163 nm for the CMNs (Figure S1). Western blot analysis further confirmed the presence of expected bands at 150 kDa for FS and 87 kDa for TS (Figure 2C). Additionally, ELISA-based quantification indicated that TS and FS accounted for 3.33% and 2.58% of the total membrane proteins, respectively (Figure 2D), demonstrating effective enrichment of both antigens on the membrane carriers.

### 3.2. Characterization of Humoral and CD8^+^ T Cell Immune Response Induced by the CMN Vaccines

**Figure 3:**
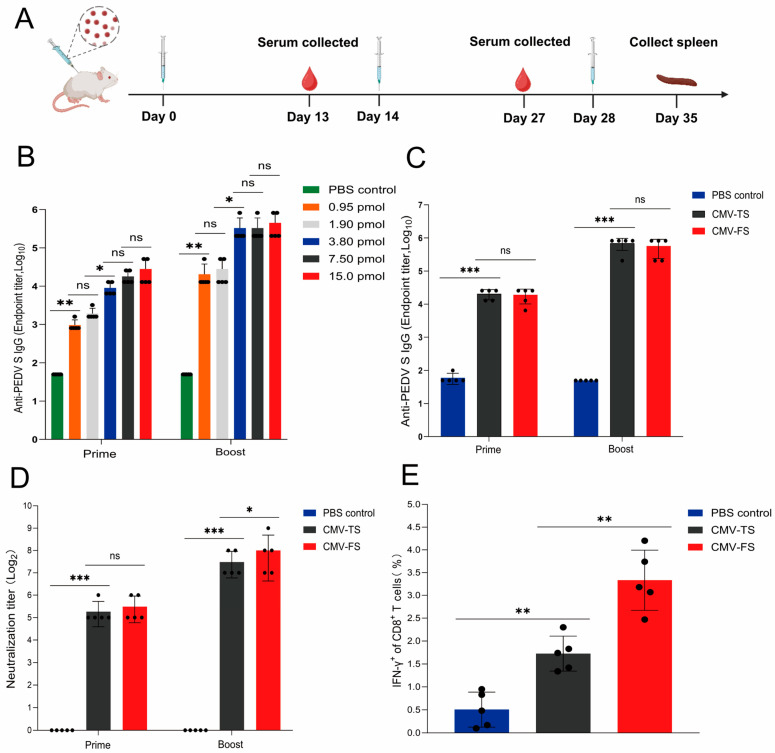
Evaluation of humoral and CD8^+^ T cell immune response induced by CMN vaccines in mice. (**A**) Schematic diagram of the immunization and immunogenicity assessment schedule. (**B**) Determination of antibody titers induced by CMN-TS vaccine at different doses. (**C**) Comparison of binding antibody titers between CMN-FS and CMN-TS vaccines. (**D**) Comparison of neutralization antibody titers between CMN-FS and CMN-TS vaccines. (**E**) Frequency of antigen-specific IFN-γ producing CD8^+^ T cells quantified by intracellular staining. (ns, not significant; * *p* < 0.05; ** *p* < 0.01; *** *p* < 0.001).

To evaluate the immunogenicity of the prepared CMN vaccines, a dose-screening experiment was first conducted. Female BALB/c mice were immunized via intramuscular injection (days 0 and 14) of CMN-TS vaccine at 0.95, 1.90, 3.80, 7.50, or 15.0 pmol (Figure 3A). Serum samples were collected 13 days after each immunization, and antigen-specific antibody levels against S protein were measured by ELISA (Figure 3B). A dose-dependent increase in antibody titers was observed up to 3.80 pmol, beyond which no further rise was detected, indicating a plateau (Figure 3B). Therefore, 3.80 pmol was selected as the optimal dose for subsequent immunization.

Using this optimized dose, we further compared the immunogenicity of CMN-FS and CMN-TS vaccines in a murine model. Mice were randomly allocated to three cohorts: CMN-FS, CMN-TS, or PBS control, and immunized on the same two-dose schedule (Figure 3A). No systemic adverse events were observed in any immunized mice, and a significant increase in antibody titers was detected after the booster immunization (Figure 3C). Specifically, there was no significant difference in the binding antibody titers between the CMN-FS and CMN-TS groups after both the initial and booster immunizations, both reaching 1:819,200. However, neutralization antibody analysis against *PEDV* GIIa strain revealed that the CMN-FS group exhibited significantly higher neutralization antibody titers (1:512) compared to the CMN-TS group (Figure 3D). These data suggest that the full-length S protein may present a more comprehensive set of functional and protective epitopes compared to the truncated S protein against the *PEDV* GIIa strain.

To further evaluate CD8^+^ T cell immune responses, splenic lymphocytes were isolated and restimulated in vitro with 10 µg/mL recombinant PEDV S protein. Additionally, the proportions of T-cell subsets were analyzed using flow cytometry. The results indicated that the proportions of IFN-γ^+^CD8^+^ T cells in both the CMN-FS and CMN-TS vaccine groups were significantly higher than those in the PBS control group. Moreover, IFN-γ^+^CD8^+^ T cells in the CMN-FS group were markedly increased compared to the CMN-TS group (Figure 3E and Figure S2). Collectively, the CMN-FS demonstrated superior immunogenicity compared to the CMN-TS vaccine, eliciting robust humoral and CD8^+^ T cell immune responses. Therefore, it represents a promising candidate for further development as a nanovaccine platform.

### 3.3. Safety Evaluation of the CMN Vaccine

**Figure 4:**
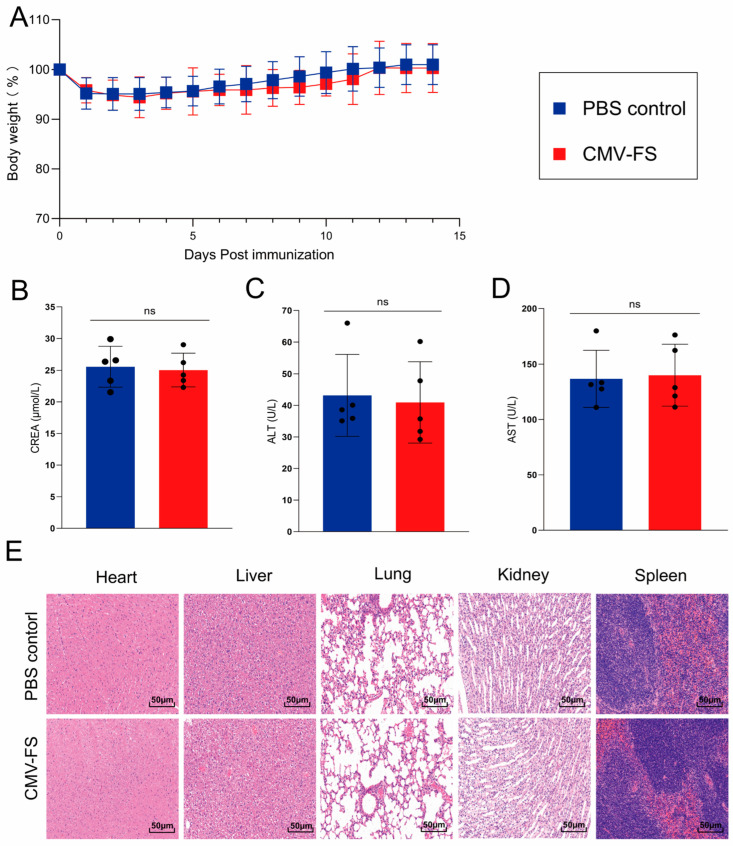
Safety evaluation of CMN-FS vaccine in mice. (**A**) Body weight changes in mice over 14 consecutive days following vaccination. (**B**–**D**) Assessment of liver and kidney function based on blood biochemical parameters: (**B**) renal function as indicated by serum creatinine (CREA); (**C**,**D**) liver function as reflected by alanine aminotransferase (ALT) and aspartate aminotransferase (AST) levels. (**E**) Histopathological analysis by hematoxylin and eosin (H&E) staining of major organs (heart, liver, lung, kidney, and spleen) from immunized mice; representative images are shown. (ns, not significant).

Following the identification of CMN-FS (3.80 pmol) as the optimal group in Figure 3, mice immunized with this formulation were subjected to a systematic, multidimensional in vivo safety evaluation of the CMN vaccine. In the dynamic body weight monitoring (Figure 4A), although a transient decrease in body weight was observed in the vaccinated group within 14 days after the initial immunization, the subsequent weight change trend was largely consistent with that of the control group (non-immunized group), with no persistent abnormal fluctuations, preliminarily indicating that the vaccine did not significantly disrupt the overall physiological state of the mice. To further assess the potential impact of the vaccine on the function of vital organs, serum samples were collected 48 h after the booster immunization for liver and kidney function-related biomarker analysis. The results showed that kidney function indicators (CREA) and liver function indicators (ALT and AST) in the vaccinated group all remained within normal physiological ranges, with no statistically significant differences compared to the control group, indicating that the vaccine caused no adverse effects on kidney or liver function (Figure 4B–D). Furthermore, histopathological examination of key organs (heart, liver, lung, kidney, and spleen) from the vaccinated group revealed intact tissue morphology, regular cellular arrangement, and an absence of pathological alterations such as inflammatory infiltration, cell necrosis, or tissue damage (Figure 4E). Collectively, these findings from body weight monitoring, organ function tests, and histopathological evaluation conclusively demonstrate that the CMN-FS possesses a favorable in vivo safety profile.

### 3.4. Immunogenicity Comparison of CMN-FS and Commercial Vaccine

**Figure 5:**
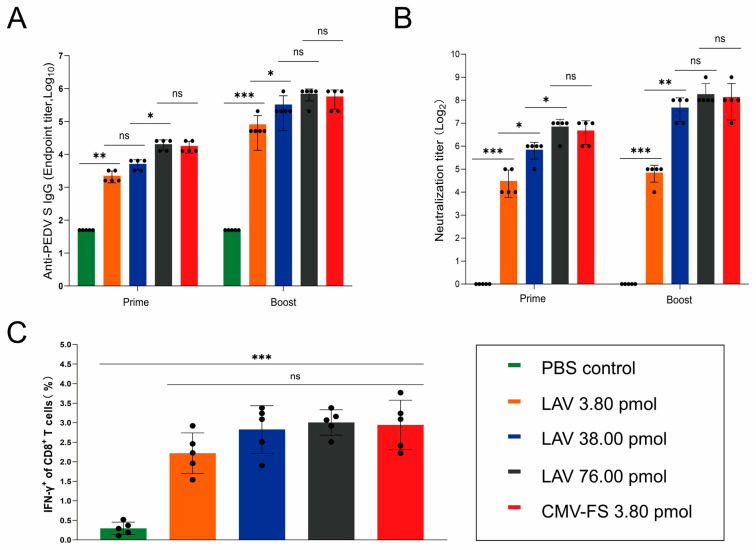
CMN-FS vaccine elicits more efficient humoral and CD8^+^ T cell immune response than the LAV vaccine. (**A**) Comparison of binding antibody titers between CMN-FS and LAV (live attenuated vaccine) at different doses. (**B**) Comparison of neutralizing antibody titers between CMN-FS and LAV. (**C**) Frequency of antigen-specific IFN-γ producing CD8^+^ T cells quantified by intracellular staining. (ns, not significant; * *p* < 0.05; ** *p* < 0.01; *** *p* < 0.001).

Given that the CMN-FS (3.80 pmol) group was identified as the optimal immunization group, we further evaluate the differences in immunogenicity between the self-developed CMN vaccine (CMN-FS, 3.80 pmol) and a commercially available vaccine (live attenuated vaccine, LAV) under the principle of “S protein dose equivalence”. Following quantification of the S protein in the commercial LAV vaccine via ELISA, we prepared corresponding groups with equivalent (3.80 pmol), 10-fold (38.00 pmol), and 20-fold (76.00 pmol) molar doses for comparison with the CMN-FS (3.80 pmol) group. Female BALB/c mice were subjected to an intramuscular immunization regimen identical to that depicted in Figure 3A. Serum samples and splenocytes were harvested at predefined time points for humoral and cellular immunological analyses. The results showed that all experimental cohorts exhibited a marked increase in antigen-specific IgG titers (Figure 5A). Notably, the CMN-FS vaccines after both prime and booster immunizations were higher than those in the 3.80 pmol commercial vaccine group and were comparable to those in the 38.00 pmol commercial vaccine group (Figure 5A). In terms of neutralizing antibody responses, the titers in the CMN-FS group were similar to those in the 38.00 pmol commercial vaccine group and even reached levels comparable to the 76.00 pmol group (Figure 5B). Furthermore, the proportion of IFN-γ^+^CD8^+^ T cells in the CMN-FS group was significantly higher than that in the commercial vaccine group with equivalent molar amounts of S protein (Figure 5C). In conclusion, the CMN-FS achieved immunogenic effects comparable or even superior to those of higher-dose commercial vaccines at a lower dosage, demonstrating a “low-dose, high-efficacy” advantage in immunogenicity.

### 3.5. Evaluations of Humoral and CD8^+^ T Cell Immune Responses Induced by the CMN-FS in Piglets

**Figure 6:**
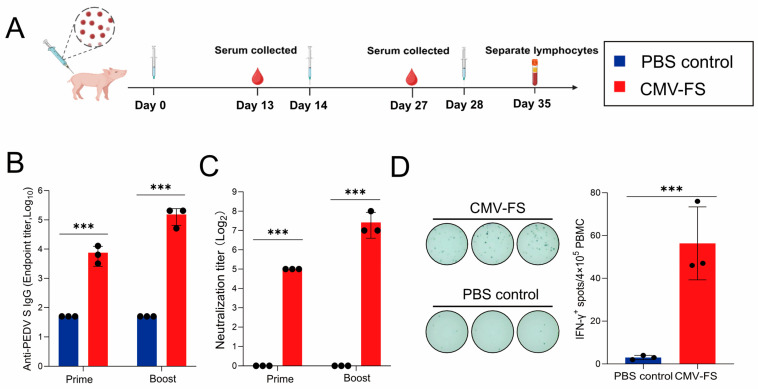
Evaluation of humoral and CD8^+^ T cell immune responses to the CMN-FS vaccine in piglets. (**A**) Schematic diagram of the immunization and immunogenicity assessment schedule. (**B**) PEDV S-specific IgG antibody titers in serum, assessed by ELISA. (**C**) Neutralizing antibody titers in serum. (**D**) Frequency of antigen-specific IFN-γ producing CD8^+^ T cells quantified by ELISPOT. (*** *p* < 0.001).

We further evaluated the immunogenicity of the CMN-FS vaccine in piglets, the natural host of PEDV. The experimental design was illustrated in Figure 6A. Piglets were divided into CMN-FS group and PBS control group. A booster immunization was given to both groups on day 14 and day 28 after the primary immunization. No adverse reactions were observed in any piglets during the vaccination process. Serum samples were collected on day 13 and day 27 to measure specific and neutralizing antibody levels. The results showed a significant increase in FS-specific antibody titers following the booster immunization (Figure 6B). Notably, high levels of neutralizing antibodies (titers ≥ 1:128) were induced in the vaccinated piglets (Figure 6C). On day 35 after the primary immunization, peripheral blood mononuclear cells (PBMCs) were isolated and restimulated in vitro with recombinant PEDV S protein to evaluate CD8^+^ T cell immune responses. As shown in Figure 6D, the proportion of IFN-γ^+^ CD8^+^ T cell was significantly higher in the CMN-FS vaccinated group compared to the PBS control group. In conclusion, the CMN-FS elicits robust humoral and CD8^+^ T cell immune responses in piglets.

## 4. Discussion

*PEDV*, as an emerging and re-emerging α-coronavirus, poses a major threat to piglet health and has inflicted substantial economic losses on the global swine industry. The development of effective vaccines against *PEDV* remains a cornerstone of disease control strategies. In this study, we engineered a CMN-based PEDV vaccine that elicits robust immune responses, offering a novel strategy for next-generation PEDV vaccine development.

Currently, the predominant genotype of *PEDV* in China belongs to the GII group. In the present study, the S protein from the prevalent GIIa strain of PEDV was initially used as the protective antigen. The results showed that no significant difference in antigen-specific IgG titers was observed between the CMN-FS and CMN-TS vaccines (Figure 3C), while CMN-FS elicited significantly superior neutralizing activity than CMN-TS (Figure 3D). Epitope coverage maybe the main reason: compared to TS, the FS likely presents a more comprehensive repertoire of functional and protective epitopes. However, cross-protection against other subtypes (e.g., GIIb, GIIc) was not evaluated here; prior reports indicate that GIIa-based vaccines may provide limited cross-protection [25,26]. Nevertheless, the CMN platform can be rapidly updated by replacing or co-expressing antigens, facilitating the design of broadly protective vaccines against diverse PEDV variants.

Preserving the native conformational integrity of antigens is a critical prerequisite for inducing potent neutralizing antibodies [27]. Conventional inactivated vaccines, which rely on formaldehyde cross-linking, often disrupt the conformational epitopes of the PEDV S protein, resulting in limited cross-protection [28]. Although live attenuated vaccines can elicit both mucosal and cellular immunity by mimicking natural infection, they carry a non-negligible risk of virulence reversion [9]. This potential hazard necessitates stringent biosecurity monitoring systems on farms, substantially increasing the complexity of vaccine deployment. Subunit vaccine development faces inherent limitations in terms of expression systems. For instance, prokaryotically expressed PEDV S protein often forms inclusion bodies, and after refolding, significantly impairs the induction of neutralizing antibodies [29]. Although eukaryotic systems (e.g., CHO cells, yeast) enable glycosylation, the heterogeneity in glycosylation patterns across host cells leads to batch-to-batch variability in antigen structure, compromising immune response consistency [30]. While emerging mRNA vaccines can provoke humoral and cellular immunity, they exhibit poor stability and are highly dependent on cold-chain transportation [20]. In contrast, the CMN technology preserves the native conformation of protein while mimicking the bilayer phospholipid structure of the viral envelope [27,31,32], thereby significantly enhancing antigen presentation efficiency [21]. Our results demonstrate that a dose as low as 3.80 pmol of the CMN-FS vaccine induced antibody titers comparable to those achieved with 38.00 pmol of a commercial vaccine, with neutralization antibody levels approaching those of the high-dose group (76.00 pmol). Furthermore, by presenting antigens through natural membrane anchoring, our platform eliminates the risk of virulence reversion associated with live attenuated vaccines and closely mimics the native antigen structures.

In terms of production workflow, the present vaccine platform also demonstrates notable advantages. Unlike traditional vaccine production, it eliminates the need for cumbersome procedures such as formaldehyde treatment, thermal inactivation, or irradiation to abolish pathogen infectivity [33,34,35]. It also avoids the dependence on reverse genetics systems for constructing viral vector vaccines (e.g., VSV-based vectors), which involve extended production timelines and require Biosafety Level-2 (BSL-2) facilities, thereby increasing biosafety concerns [36,37]. Furthermore, the platform circumvents the cold-chain dependency and storage challenges associated with mRNA vaccines [20]. Additionally, this vaccine platform employs a “physical lysis–gradient centrifugation” process to generate non-infectious antigens. This approach fundamentally bypasses complex inactivation steps, thereby preventing potential structural damage or loss of immunogenicity caused by inconsistent inactivation conditions.

In summary, the CMN vaccine developed in this study elicits robust immune responses, representing a strategy for *PEDV* control that integrates scientific innovation with translational potential. In piglet models, booster immunization induced not only notable neutralizing antibodies but also elevated frequencies of IFN-γ^+^ CD8^+^ T cells. It should be noted that our current CMN platform is based on Expi293F cells, which may introduce non-antigen-related membrane proteins and raise potential safety and unexpected concerns. To address this, we plan to adopt ST cells (porcine testis cells) for vaccine production, as their porcine origin reduces the risk of species-specific immune reactions. Future studies will include pig challenge experiments to evaluate protective efficacy and investigations into cross protection against heterologous strains. Given its versatility, this platform could also be extended to multivalent vaccine formulations (e.g., against *PEDV*, *TGEV*, and *PDCoV* simultaneously), providing a promising strategy for developing next generation veterinary vaccines.

## Data Availability

The original contributions presented in this study are included in the article. Further inquiries can be directed to the corresponding author.

## Supplementary Materials

The following supporting information can be downloaded at https://www.mdpi.com/article/10.3390/cells15020208/s1, Figure S1: Size distribution of the CMNs. Size distribution of the CMNs was measured by a Nanoparticle Tracking Analyzer; Figure S2: Flow cytometric gating strategies for the identification and quantification of IFN-γ^+^ CD8^+^ T cell subset. Representative flow cytometry plots depict the sequential gating strategy: Lymphocytes were first defined by FSC-A and SSC-A, followed by gating of single cells via FSC-A and FSC-H, followed by selection of live cells with eFluor450. From the live lymphocytes, CD8^+^ T cells were identified, and IFN-γ producing cells were further gated.
