# Supplementary material for: Development of a Cellular Membrane Nanovesicle-Based Vaccine Against Porcine Epidemic Diarrhea Virus
Source: Cells. 2026 Jan 22;15(2):208. doi: 10.3390/cells15020208 (PMC12840353; doi:10.3390/cells15020208)
Supplement: Supplementary file 1 [file cells-15-00208-s001.zip › cells-4031262-supplementary.pdf]

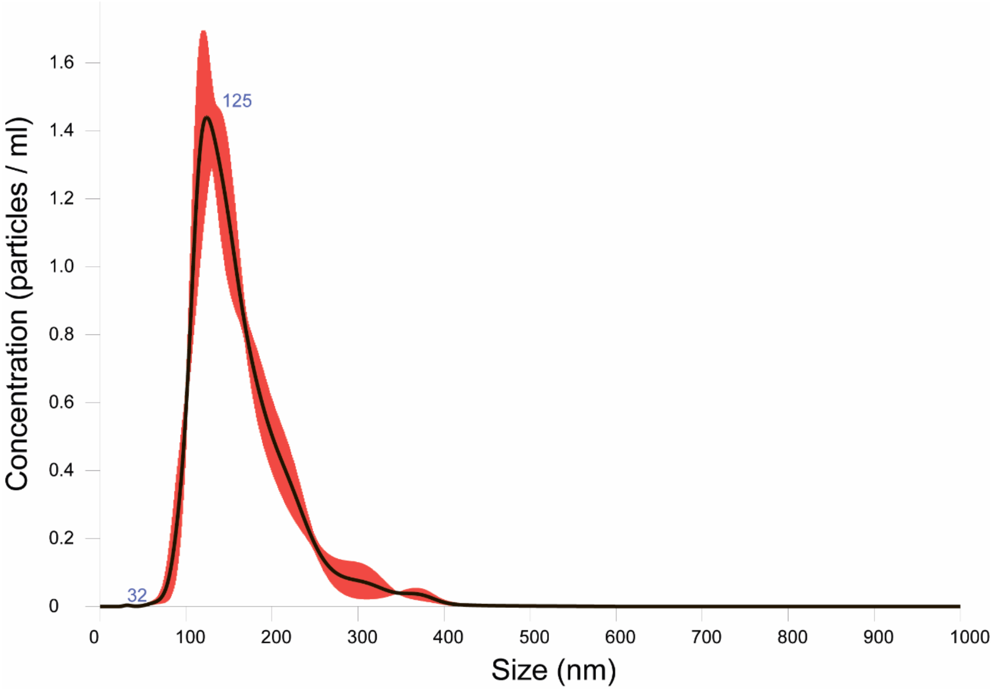

Figure S1. Size distribution of the CMNs. Size distribution of the CMNs was measured by a Nanoparticle Tracking Analyzer.

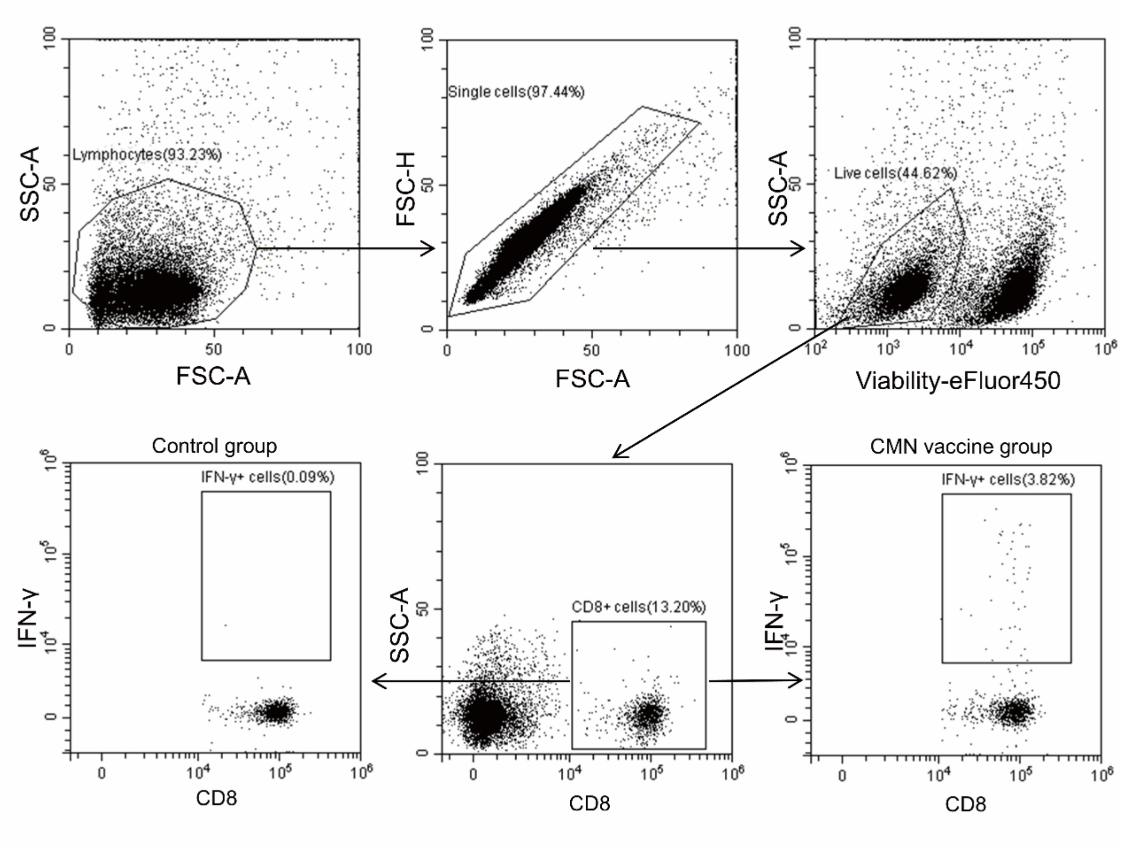

Figure S2. Flow cytometric gating strategies for the identification and quantification of IFN-γ^{+} CD8^{+} T cell subset. Representative flow cytometry plots depict the sequential gating strategy: Lymphocytes were first defined by FSC-A and SSC-A, followed by gating of single cells via FSC-A and FSC-H, followed by selection of live cells with eFluor450. From the live lymphocytes, CD8^{+} T cells were identified, and IFN-γ producing cells were further gated.
